# NiO Nanofibers as a Candidate for a Nanophotocathode

**DOI:** 10.3390/nano4020256

**Published:** 2014-04-03

**Authors:** Thomas J. Macdonald, Jie Xu, Sait Elmas, Yatin J. Mange, William M. Skinner, Haolan Xu, Thomas Nann

**Affiliations:** Ian Wark Research Institute, University of South Australia, Mawson Lakes 5095 SA, Australia; E-Mails: thomas.macdonald@mymail.unisa.edu.au (T.J.M.); jie.xu@mymail.unisa.edu.au (J.X.); sait.elmas@unisa.edu.au (S.E.); yatin.mange@mymail.unisa.edu.au (Y.J.M.); bill.skinner@unisa.edu.au (W.M.S.); haolan.xu@unisa.edu.au (H.X.)

**Keywords:** Nickel oxide, nanofibers, photoelectrode, photocathode, electrospinning

## Abstract

*p*-type NiO nanofibers have been synthesized from a simple electrospinning and sintering procedure. For the first time, *p*-type nanofibers have been electrospun onto a conductive fluorine doped tin oxide (FTO) surface. The properties of the NiO nanofibers have been directly compared to that of bulk NiO nanopowder. We have observed a *p*-type photocurrent for a NiO photocathode fabricated on an FTO substrate.

## 1. Introduction

Nickel oxide (NiO) is an attractive semiconductor, because its properties are very useful for various photocatalytic [1], battery [2], electrochromic and chemical sensing [3,4] applications. It has been previously recognized that altering the morphology of NiO changes the performance of a device [5]. Recent studies have been conducted to investigate the performance of different NiO structures [1,6,7]. Among the various morphologies, the best performance was found to be those that exhibit a high specific surface area, in particular nanofibers [4]. While a majority of the work on photovoltaic devices uses metal oxide films (for example, titania (TiO_2_) thin films), a new approach employs high surface area nanofibers. One-dimensional (1D) TiO_2_ metal oxide nanofibers have been previously reported and applied for solar cell applications [1,2,5,8]. Nanofibers have the advantage of having a high surface area developed through the pores and grains within the fibrous structure. Furthermore, 1-D nanofibers ensure rapid collection of charge carriers, very beneficial for photovoltaic devices [9]. Nanofibers also combine small primary particle size with high porosity, resulting in surface areas several orders of magnitude higher than regular films [10].

Electro-spun nanofibres are usually prepared using a metal containing sol-gel polymer solution to support and shape the fibers. Electrospinning uses an electrical charge to draw very fine fibers from a liquid. When a high voltage is applied to a liquid droplet, the body of the liquid becomes charged and electrostatic repulsion counteracts the surface tension causing the droplet to be stretched. When the liquid is stretched, it forms a stream of liquid that erupts from the needle’s tip. The liquid dries in flight and is elongated by electrostatic repulsion initiated at small bends of the fiber until it is finally deposited on the ground collector. Polymers are then burnt-off along with any other unwanted volatile precursors and the metal oxides are sintered. For this to occur an understanding of the thermal properties of the system is essential. While the conversion of Ni(OH)_2_ to NiO occurs at 250 °C, Boshloo and Hagfeldt illustrated that at this temperature, cubic NiO (bunsenite) is formed [10]. While their study investigated the formation of NiO nanostructures using various salt precursors they state that the formation of NiO nanostructures does not occur until 300–320 °C. Later studies commonly sinter Ni(OH)_2_ species closer to 500 °C to achieve nanostructures, because the decomposition of most polymers or salt precursors, occurs around 400–500 °C [1,5,6,10,11]. Fu *et al.* [1] reported the formation of porous NiO nanosheets for use in *p*-type photoelectrodes for dye sensitized solar cells (DSSCs) [1]. They also report on the formation of two different types of crystal structure phases, α, and β for NiO. The α phase is generally unstable and obtained at lower temperatures, for Ni(NO_3_)_2_ this occurs around 170 °C. Fu *et al.* [1] fabricated β NiO nanosheets without impurities at 400 °C, supporting the high temperature sintering of NiO [1].

In photovoltaic devices, it is especially important to harvest as much light as possible by producing a large *p*/*n* heterojunction. By going from a flat surface to a 3-D surface increases the area for light harvesting while simultaneously gaining a large degree of porosity from the layers of nanofibers. As a result, these types of electrodes enhance the excitation (hole/electron formation) as well as the mobility of charge carriers, hence increases the efficiency of devices [4]. It still remains a challenge to produce an efficient NiO photocathode so by introducing nanofibers. We hope to contribute to the current research and help improve the efficiencies of photocathode devices. In order to harvest more light, this may be achieved by increasing the surface area and/or sensitizing metal oxides with organic dyes or quantum dots (QDs).

TiO_2_ is the most studied sensitized metal oxide material for use in photovoltaic devices, particularly for highly efficient dye-sensitized solar cells [12,13] and water-splitting photoelectrochemical cells (PEC) [14,15]. While mesoscopic TiO_2_ films have been under extensive investigation since the discovery of the Grätzel cell [12], the most efficient TiO_2_ based DSSC had an efficiency of 12% [13], which was achieved with a cobalt (II/III)- based redox electrolyte. While the above-mentioned efficiencies are commonly reached using expensive Pt counter electrodes, NiO has recently shown to be a promising semiconductor for use in tandem solar cells (TSC) which essentially replaces platinum [1,16,17]. Although NiO has recently shown to be a promising candidate for a photocathode, fabricating a photocathode, which performs to the same level as a TiO_2_, remains a challenging process. While tandem dye-sensitized solar cells can provide a route to highly efficient energy conversion, the biggest challenge is finding a *p*-type photocatalyst comparable to that of TiO_2_. This is because it is difficult to match the current generated at the photocathode to that of the anode. Lindquist *et al.* [18] first proposed that by replacing the platinum counter electrodes in Grätzel cells with a *p*-type photocathode should greatly improve the device efficiency by collecting high energy photons at the top of the electrode and low energy photons at the bottom [18,19]. While this was the first demonstration of a cathodic current, a few other sensitizers including coumarin [8], perylene imide [20], and derivatives of prophyrin [17] have been used.

Some recent breakthroughs with *p*-type NiO photocathodes in tandem solar cells include that by Hammarstrom *et al.* [8], where they achieved an open circuit potential (*V*_OC_) of 0.91 V, and an efficiency of 0.55%. More recently, Powar *et al.* [7] achieved a *V*_OC_ of 0.79 V but an efficiency of 1.3%, which can be mainly attributed to an impressive short circuit photocurrent (*J*_SC_) of 4.44 mA/cm^2^.

While numerous studies for the sensitization of NiO with organic dyes [17] have proven to be promising in both tandem and DSSCs [8], this article focuses on the synthesis and electrode fabrication for a nanophotocathode. Rather than screen-printing or doctor blading NiO paste, this study shows the fabrication of a photocathode using only electro-spinning, without organic binders such as terpineol. The electrodes are fabricated by directly depositing the nanofibers onto fluorine doped tin oxide (FTO) that exhibits diode characteristics.

## 2. Results and Discussion

### 2.1. Structural and Surface Characterization

**Figure 1:**
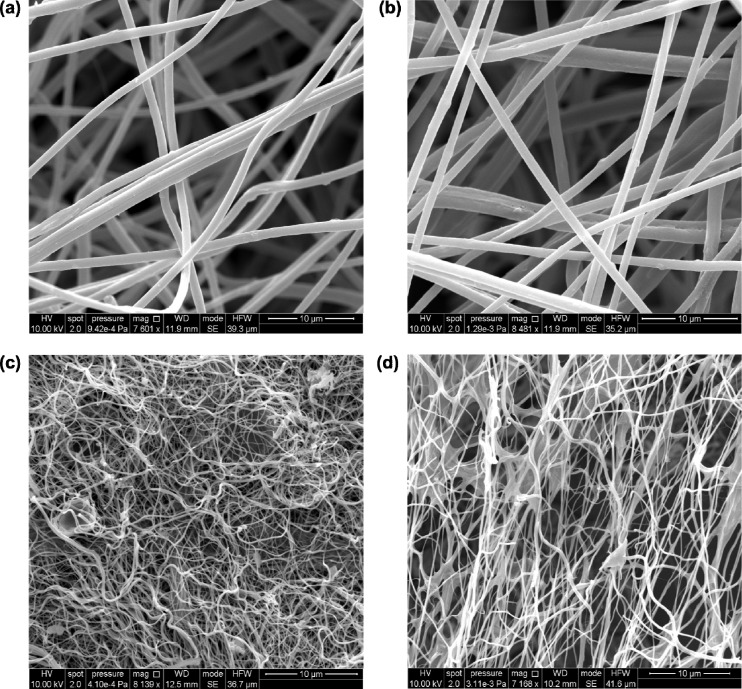
SEM for NiO nanofibers: (**a**,**b**) polyactonitrile (PAN) Ni(AcAc)_2_ nanofibers; (**c**) NiO nanofibers after calcinations; (**d**) NiO nanofibers on fluorine doped tin oxide (FTO) after calcinations.

The scanning electron microscopy (SEM) images of the polyactonitrile (PAN) Ni(acac)_2_ nanofibers with a 10 μm resolution are shown in Figure 1a,b. The nanofibers are approximately 300 nm in diameter and their morphology is smooth suggesting no secondary structures are present. The fibers interweave to form a nanofiberious network, which indicates good surface coverage. PAN was chosen as a coordinating ligand to the nickel metal centers. We assume it is leading to pre-organized structures for the nanofibers. As a qualitative analysis, energy-dispersive X-ray spectroscopy (EDXS) was measured to determine the presence of both the polymer and nickel species. Figure S1 shows the EDXS measurement for the PAN NiO nanofibers displaying a strong carbon peak from the polymer and small nickel peak. Figure 1c, represents the NiO nanofibers after calcination at 500 °C. The diameters of the nanofibers decreased to approximately 100 nm, which was expected after calcination of the polymer and residual precursor and consistent with literature [4]. Other than the change in diameter, another distinctive difference was the change in morphology. The nanofibers now appeared more straw-like and the surface of the nanofibers also increased in roughness. This suggests the smoothness, was due to the presence of the polymer. To confirm that the presence of the polymer had been significantly reduced, EDXS was measured. Figure S2 shows the EDXS confirming that after calcination, the carbon peak was significantly reduced and the nickel peak is clearly dominant. The small carbon peak is due to the presence of the carbon tape used to mount the nanofibers. Figure 1d shows the nanofibers after directly spinning them directly onto FTO. The morphology looks identical to the as-sintered nanofibers. EDXS was measured for the NiO nanofibers deposited on the FTO surface. Figure S3 shows the EDXS, which shows the counts for nickel were approximately double of that for carbon. While the presence of carbon may suggest incomplete calcination, it is more likely due to the presence of carbon tape, which was used to mount the samples as mentioned above. Oxygen was the dominant peak for the EDXS along with tin, which was expected from the FTO. Figure S4 shows the nanofibers at a scale of 2 μm supporting the measured diameters of the nanofibers.

**Figure 2:**
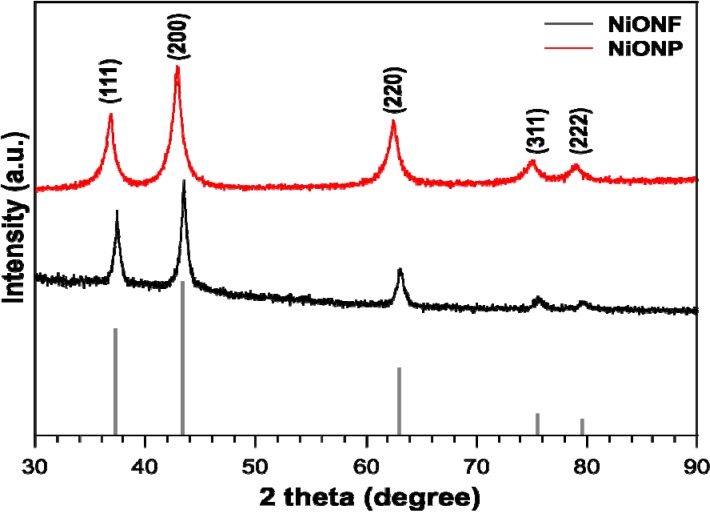
XRD for nickel oxide nanofibers (NiONF) and commercial nickel oxide nanoparticles (NiONP) [23].

NiO nanofibers were obtained by sintering PAN structured Ni(OH)_2_ nanofibers at 500 °C. The X-ray diffraction (XRD) pattern is shown in Figure 2 and is indicative of the overall crystal structure and for the synthesized NiO nanofibers. Commercial NiO nanoparticles (Aldrich) were compared to our synthesized nanofibers and revealed the same β crystalline structure. In comparison with the commercial nanoparticles, the XRD pattern for nanofibers has slightly more defined, sharp and narrow peaks indicative of high cystrallinity and a larger size [21]. Furthermore, the colour of the nanofibers was dark grey, whereas the color of the commercial nanoparticles was black. NiO species may appear anywhere from green to black depending on what temperature they are treated at. This color change has been previously recognized and attributed to a higher diffuse reflection, which provides an increase in light scattering ability provided both materials have similar specific surface areas [21]. For use in solar devices, this higher diffuse reflection is also expected to increase the optical path length, benefitting the photon-to-current conversion [19,21].

**Figure 3:**
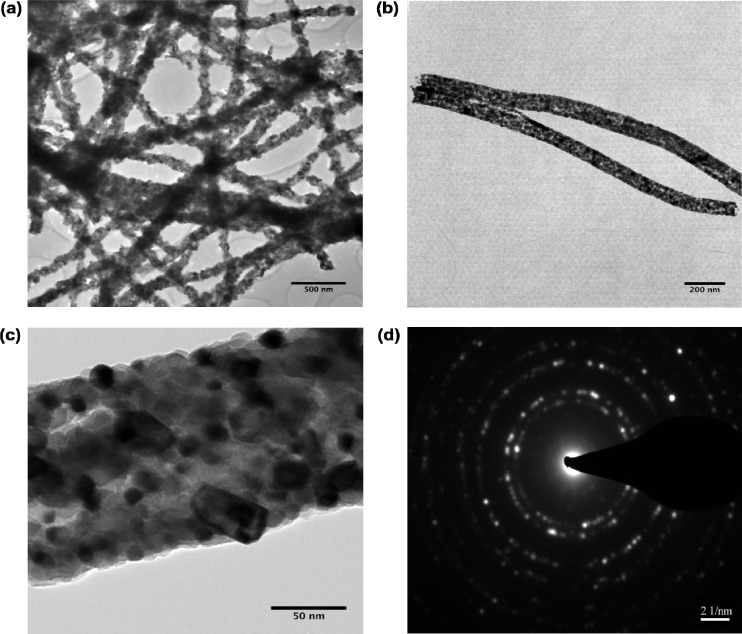
TEM for NiO nanofibers: (**a**) series of NiO nanofibers; (**b**) isolated pair of NiO nanofibers at (**c**) individual nanofiber; (**d**) selected area electron diffraction (SAED) pattern of nanofibers.

The transmission electron microscope (TEM) images of the NiO nanofibers and selected area electron diffraction (SAED) patterns are shown in Figure 3. Figure 3a shows a series of nanofibers at a 500 nm resolution. This sample was prepared by sonication of the nanofibers in ethanol before they were deposited on the TEM grid. After sonication for 30 min we can conclude that the nanofibers still maintained their fibrous morphology and were not damaged from the sonication. Figure 3b shows an isolated pair of nanofibers at a resolution of 200 nm. Figure 3c shows a higher resolution image of 50 nm where the small black dots inside the fibers suggest the individual NiO particles from which the fiber is formed. The TEM shows that the diameter of the NiO nanofibers is approximately 100–150 nm, future work will include dynamic light scattering (DLS) measurements. Figure 3d shows the SAED pattern indicating the five lattice fringes, typical of cubic NiO [21] which is in agreement with the XRD [22]. Along with the XRD in Figure 2 the sharp lattice fringes are indicative of the highly crystalline structure for the NiO nanofibers.

### 2.2. XPS

**Figure 4:**
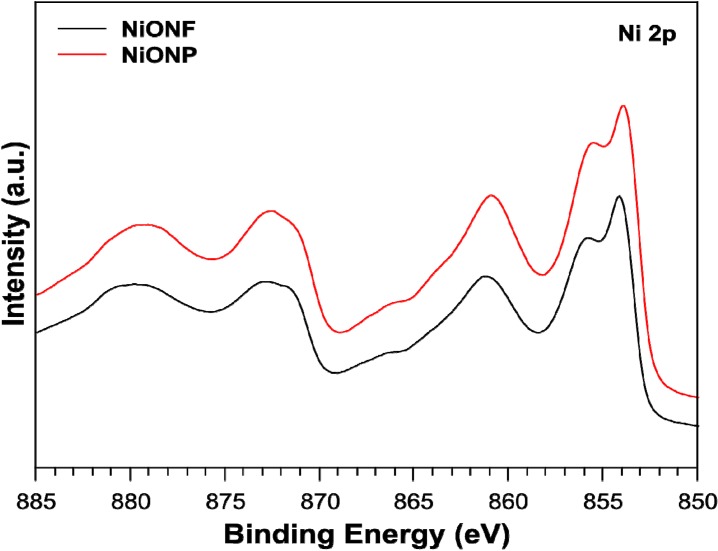
X-ray photoelectron spectroscopy (XPS) Ni 2p spectra for NiO nanofibers and nanoparticles.

**Figure 5:**
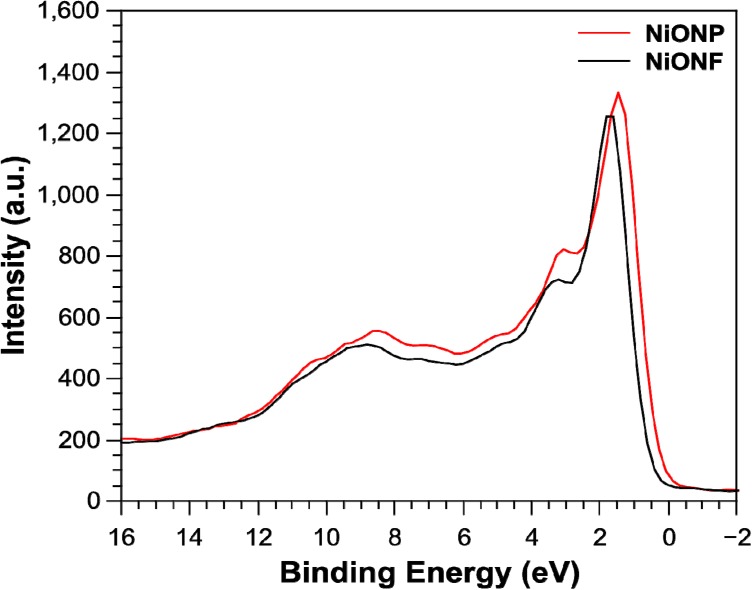
Ni valence spectra for NiO nanofibers and nanoparticles.

X-ray photoelectron spectroscopy (XPS) measurements were undertaken to investigate the chemical state of the elements in the as-synthesized products. In doing, so we looked at the Ni 2p and the Ni valence band spectra. Figure 4 shows the Ni 2p spectra where the peaks are almost identical for both the nanoparticles and the nanofibers and consistent with that observed by Li *et al.* in 2012 [24]. There are small differences in the valance band XPS spectra for NiO nanoparticles and nanofibers as shown in Figure 5. The spectra are consistent with that of work completed by Luches *et al.* in 2001 [25]. Here, nanofibers showed an apparent shift in the Fermi edge to lower binding energy, suggesting lower Ni 3d occupancy at the top of the valence band compared to the nanoparticles. This may be linked to the more uniform crystallinity indicated by the narrower XRD peak widths for the fibers *versus* particles. Despite these subtleties, both particles and fibers exhibit bulk NiO characteristics.

### 2.3. Photocurrent Response of NiO Nanoparticles

**Figure 6:**
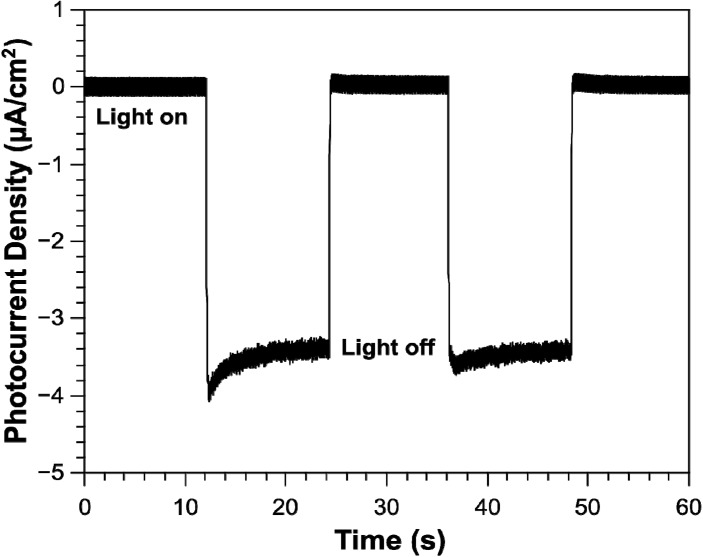
Chronoamperogram for the photocurrent as a function of time for NiO nanoparticles on FTO.

The photocurrent response of NiO nanoparticles on FTO was measured in a photo-electrochemical cell (PEC), the chronoamperogram is shown in Figure 6. Our system contained a platinum counter electrode, Ag/AgCl reference electrode and the electrolyte used was phosphate buffered saline (PBS) pH 7.4. At a bias potential of 0 V *vs.* Ag/AgCl we can clearly observe a photocurrent response in the cathodic direction. Typically, the photocurrent response for *p*-type semiconductors is negative due to the band bending at the electrode interface [26]. The bands of *p*-type semiconductors (photocathodes) bend towards lower energies, which lead to a collection of negative charge at the interface. In 2012, Mozer *et al.* [27] produced a dye-sensitized NiO photocathode for sustained hydrogen generation in a PEC. The authors also observe a cathodic current for the dye-sensitized NiO photocathode. They also find NiO to be a stable photocathode in aqueous electrolytes with a faradaic efficiency for hydrogen generation of 80%. With the recent report by Mozer *et al.* [27], we can suggest that since there is no redox mediator in our PEC system, the photocurrent response may be due to water reduction. Future work will include measuring for the presence of hydrogen, the faradaic efficiency and solar to hydrogen conversion efficiency. The surface coverage of the NiO nanofibers deposited directly onto the FTO was not dense enough to show a photocurrent response. Future work will involve fabricating a better surface deposition technique, which is denser in NiO in order to measure a photocurrent response. Although the nanofibers should provide an increased surface area and favor the rapid collection of charge carriers over commercially available nanoparticles, we must also consider the potential boundaries created by the nanofibers. The nanofibers are built upon nanograins that may also contribute to many grain boundaries, which may affect our photocurrent measurements. Further testing of the nanofibers is required before we can determine this. Although we are yet to successfully test the nanofibers on an electrode, once fabricated, we can speculate faster hole/electron transport between the working and counter electrodes due to the theoretical faster charge mobility made available by the nanofibers [4].

## 3. Experimental Section

### 3.1. Chemicals

Ninety-five percent Nickel(II) acetylacetonate (Aldrich), Polyacyrlonitrile M_W_ 150,000 (Aldrich), Nickel Oxide Nanopowder (Aldrich), 95% Terpineol (Aldrich), 48% Ethyl cellulose (Aldrich).

### 3.2. Synthesis of NiO Nanofibers

In a typical synthesis, a 5 mL solution of 12% PAN in DMF was made by slowly dissolving 0.560 g of PAN in 4.40 mL of DMF. Once dissolved, 0.250 g of Ni(AcAc)_2_ was added and the mixture was magnetically stirred for 2 h at 50 C. The solution was then loaded into a plastic syringe equipped with a 23 G stainless steel needle. The needle was then connected to a high-voltage supply (Gamma High Voltage Research, Ormand Beach, FL, USA). The solution was fed at a rate of 1.0 mL/h using a syringe pump (PHD 2000, Harvard Apparatus, Holliston, MA, USA). A piece of flat aluminum foil was placed 10 cm below the tip of the needle as a collector plate. The voltage for the electro-spinning was set to 17 kV and was conducted in a controlled temperature environment (25 °C). The fibers were electro-spun until the solution was depleted before they were then sintered at 500 °C for 2 h, yielding black NiO nanofibers sheets.

### 3.3. Fabrication of NiO Nanofiber Electrode

Following the same procedure as above 2 × 2 cm sheets of FTO (Solaronix, 15 ohm/square) were placed directly under the needle on the aluminium foil. Conductive tape was used to form a connection between the foil and the FTO. Nanofibers were directly spun onto the FTO electrode, yielding a sticky green fiberous coating. The electrodes were then sintered at 500 °C for 2 h to remove the insulating polymer, yielding a monolayer of black NiO nanofibers on the surface of the FTO.

### 3.4. Fabrication of NiO Particle Electrode

NiO nanoparticle slurry was made according to literature [17,28]. Briefly, three grams of NiO nanoparticles (Alrich) were mixed with a 10% ethyl cellulose solution in 20 mL of terpineol. The solution was mixed overnight to ensure the particles were well dispersed. Ethanol was then slowly removed by rotary evaporation. The NiO slurry was then applied to 2 × 2 cm sheets of FTO (Solaronix, 15 ohm/square) by doctor blade technique. The surface area of the fabricated photocathode was 0.8 cm^2^. Finally, the electrode was sintered at 450 °C for 30 min to remove all organics.

### 3.5. Photocurrent Measurements

These measurements used an Ag/AgCl reference electrode, platinum counter electrode and phosphate buffered saline (PBS) electrolyte (pH 7.4) in a photoelectrochemical cell (PEC). The PEC was illuminated with 100 mW/cm^2^ AM 1.5 radiation (Solar Simulator 2000, Abet Technologies, Milford, CT, USA).

## 4. Conclusions

In conclusion, we have successfully fabricated a *p*-type nanophotocathode from NiO nanofibers from a simple electrospinning and sintering procedure. For the first time, *p*-type nanofibers have been electrospun onto a conductive FTO surface. While the surface coverage did not provide us with a reproducible photocurrent, future work is to explore other means of nanofiber surface deposition to increase photocurrent response.

## Supplementary Materials

Supplementary File 1
