# Supplementary material for: NiO Nanofibers as a Candidate for a Nanophotocathode
Source: Nanomaterials (Basel). 2014 Apr 3;4(2):256–66. doi: 10.3390/nano4020256 (PMC5304677; doi:10.3390/nano4020256)
Supplement: Supplementary File 1 [file nanomaterials-04-00256-s001.docx]

Supplementary Information

**Figure S1.** EDXS for PAN NiO(acac)_2_ nanofibers.


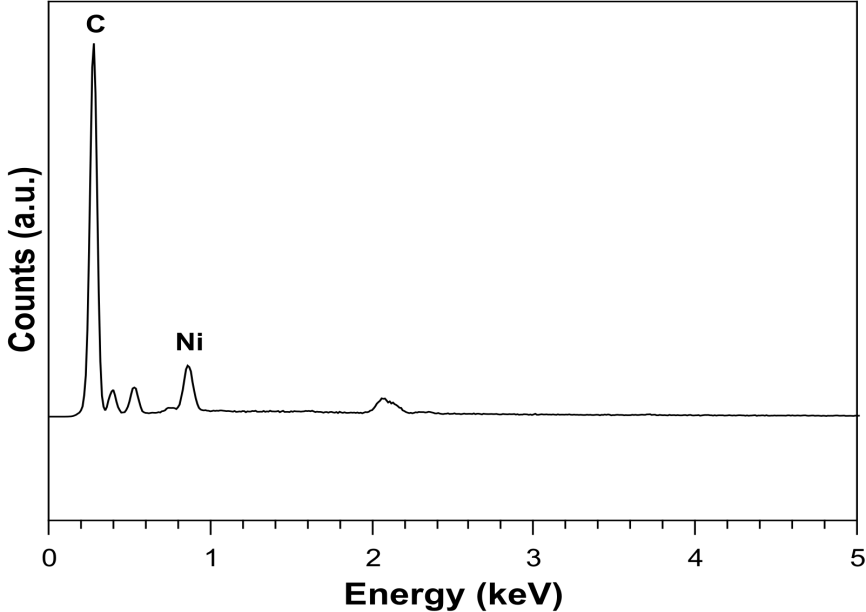


**Figure S2.** EDXS for NiO nanofibers.


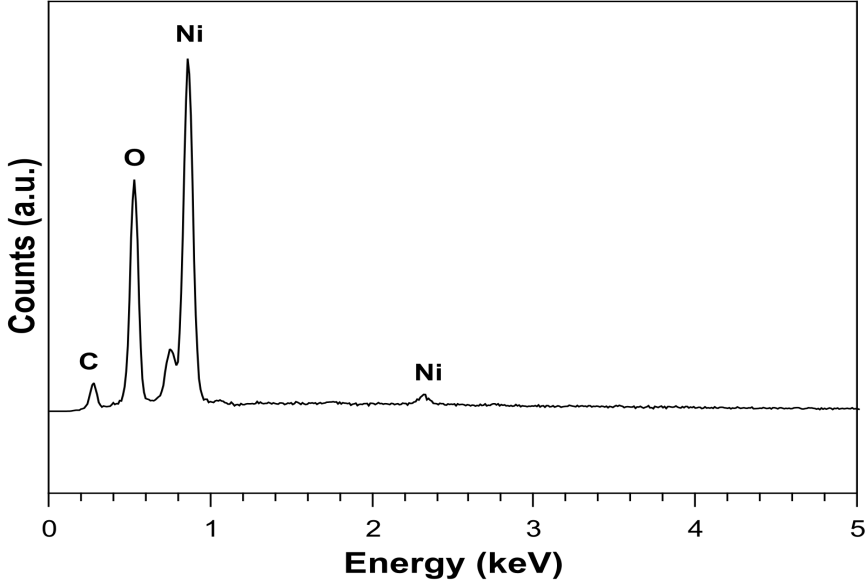


**Figure S3.** EDXS for NiO nanofibers on FTO.


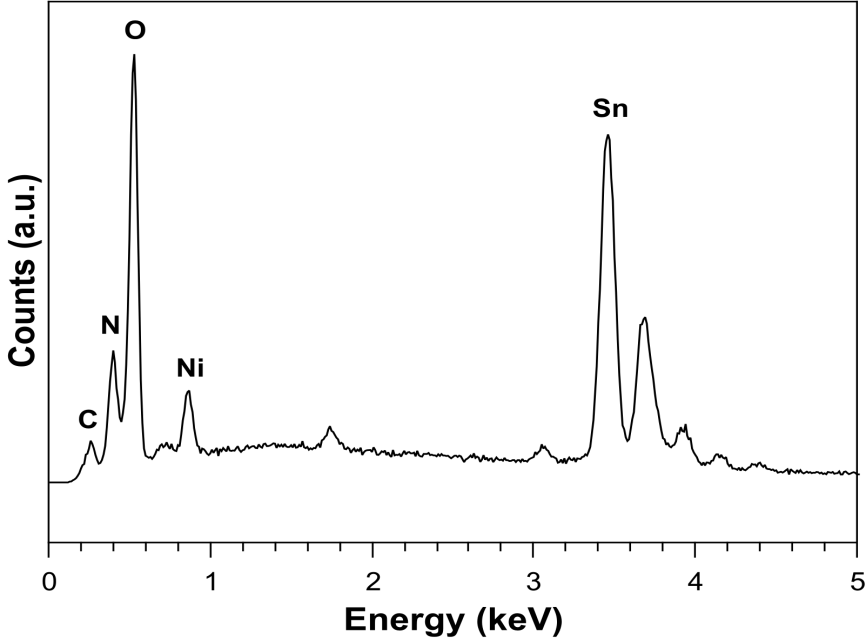


**Figure S4.** 2 μm SEM for NiO nanofibers after calcinations.


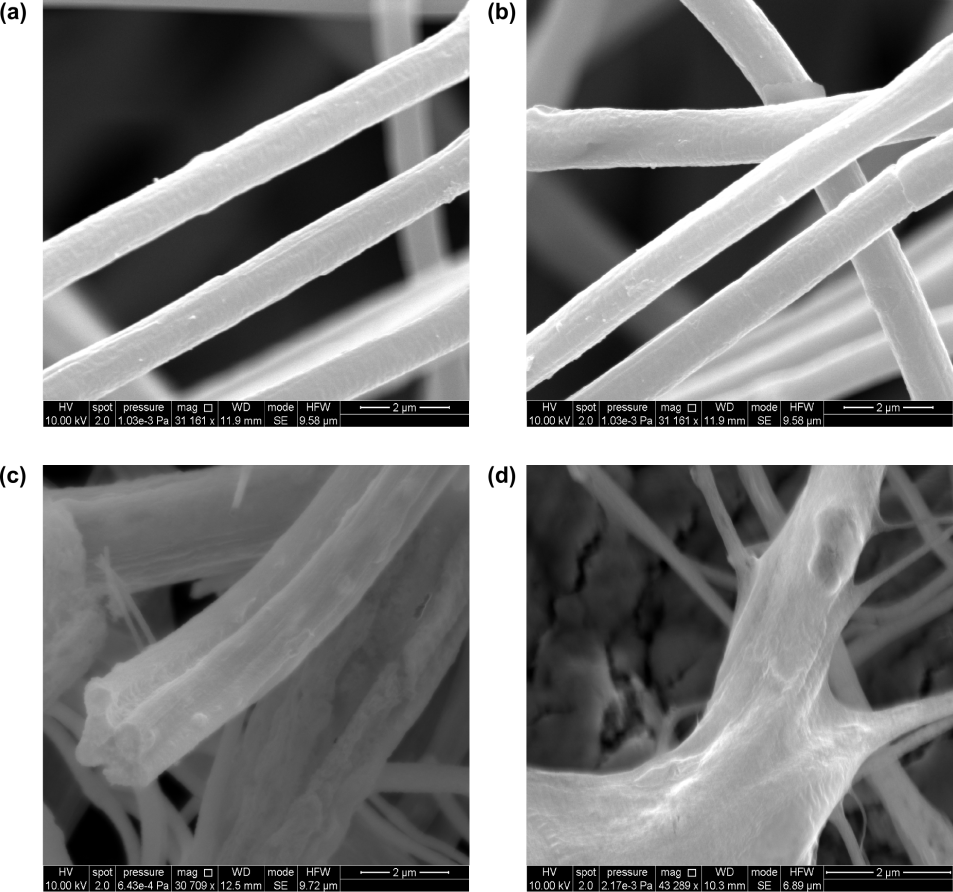


© 2014 by the authors; licensee MDPI, Basel, Switzerland. This article is an open access article distributed under the terms and conditions of the Creative Commons Attribution license (http://creativecommons.org/licenses/by/3.0/).
